# 531 Comparison of Clinical Estimation and Stereophotogrammic Instrumented Imaging of Burn Scar Height and Volume

**DOI:** 10.1093/jbcr/irac012.160

**Published:** 2022-03-23

**Authors:** Shyla K Bharadia, Vincent Gabriel

**Affiliations:** University of Calgary, Calgary, Alberta; University of Calgary, Calgary, Alberta

## Abstract

**Introduction:**

Descriptive clinical tools for the characterization of burn scar features are limited by variability between users and unknown sensitivity to change over time. We have previously described pre-clinical assessment of stereophotogrammetry as a valid measure of burn related scar and in this study compare the estimated vs. instrumented measurement of maximum height and total positive volume of burn scars in a tertiary care adult outpatient burn clinic.

**Methods:**

This study was approved by our university’s research ethics board. All participants provided written informed consent. Persons 18 years or older presenting to an outpatient burn clinic with closed burn scar that may be captured in a single image were enrolled in the study. Patients with scars from other injuries or who were unable to provide consent were excluded. Photographs of burn scars were taken with a commercially available 3D camera. Three experienced wound care therapists estimated the maximum height and total positive volume of the collected images. The images were assessed with stereophotogrammic software with results exported to a spreadsheet for further analysis. Two factor analysis without replication was performed to calculate intra-class correlation coefficients (ICC) between the assessors estimated scar height and volume and the measured height and volume. Two sided Wilcoxon tests were performed comparing mean estimated height and volume between estimated and measured output.

**Results:**

Fifteen participants with a mean age of 42.6 (21-68) were enrolled. Twenty-six scar images were taken from wounds that were managed by non operative treatment (20), excision and grafting (5), and 1 image was collected from a skin graft donor site. Scar images were taken of the trunk and extremities, but none of the head nor neck. The estimated maximum scar height ICC was 0.595 and volume 0.531. The measured scar height ICC was 0.933 and volume 0.890. Wilcoxon tests of estimated and measured volume were significantly different (z = -2.87, p = 0.041). Comparison of estimated and measured height were not significant (z = -1.39, p 0.161).

**Conclusions:**

Stereophotogrammic measurement of maximum scar height and total scar volume is more reliable than clinical photograph assessment. Clinical estimation of scar volume is significantly less than instrumented measurement, although maximum estimated vs. measured scar height was not significant in this study. There are limitations in measuring scar properties for image capture that exceeds the visual field.

